# Trends and Future Prospects of the Drowsiness Detection and Estimation Technology

**DOI:** 10.3390/s21237921

**Published:** 2021-11-27

**Authors:** Toshiya Arakawa

**Affiliations:** Department of Information Technology and Media Design, Nippon Institute of Technology, Miyashiro-Machi, Saitama 345-0826, Japan; arakawa.toshiya@nit.ac.jp; Tel.: +81-480-34-4111

**Keywords:** drowsiness, driver monitoring, biometric information, vehicle behavior, graphic information, autonomous driving

## Abstract

Drowsiness is among the important factors that cause traffic accidents; therefore, a monitoring system is necessary to detect the state of a driver’s drowsiness. Driver monitoring systems usually detect three types of information: biometric information, vehicle behavior, and driver’s graphic information. This review summarizes the research and development trends of drowsiness detection systems based on various methods. Drowsiness detection methods based on the three types of information are discussed. A prospect for arousal level detection and estimation technology for autonomous driving is also presented. In the case of autonomous driving levels 4 and 5, where the driver is not the primary driving agent, the technology will not be used to detect and estimate wakefulness for accident prevention; rather, it can be used to ensure that the driver has enough sleep to arrive comfortably at the destination.

## 1. Introduction

The National Highway Traffic Safety Administration (NHTSA) estimated that drowsy driving accounted for 91,000 traffic accidents, which caused approximately 50,000 injuries and 800 deaths, as reported by the police in 2017. However, individuals in the fields of traffic safety, sleep science, and public health have unanimously agreed that these figures underestimate the impact of drowsy driving [1]. The National Sleep Foundation reports that 54% of adult drivers feel drowsy while driving, and 28% have attested that they fall asleep while driving. In addition, more than 40% admit falling asleep at the wheel at least once while driving [2].

Drowsiness is considered a transition from arousal to sleep [3]. The first sign of drowsiness is the inability to keep one’s eyes open. Frequent closing of one’s eyes makes it impossible to perform their task effectively. Next, one’s head tends to be shaken back and forth when they are drowsy. Yawning is also a sign of drowsiness [4]. Driving when one is sleep-deprived is similar to drunk driving. As one feels sleepier, the response time increases, the ability to foresee danger decreases, and the durability of attention decrease. Driving with no sleep for more than 20 h is equivalent to driving with a blood-alcohol level of 0.08%, which is the legal limit in the United States. Microsleeps (short periods of unconscious inattention) may also occur in some people [5]. The driver’s state is related to various psychological, physical, and mental factors [6]. Particularly, fatigue and monotony are considered to decrease attention and arousal levels, resulting in drowsiness [6]. Drowsiness can lead to accidents at any time of the day or night. Such accidents due to drowsiness have the following characteristics [1]:

1. Often occurs between midnight and 6 a.m. or in the late afternoon. During both times, the circadian rhythm, the body’s internal clock that controls sleep, is reduced;
2. In many cases, a single driver (without a passenger) has run off the road at high speed with no sign of braking;
3. Often occurs on local roads and highways.

In some situations, one may be involved in any of these cases. Therefore, one needs to be careful to avoid being drowsy, not drive when drowsy, and have enough rest when they feel drowsy. However, one might still drive when drowsy. Thus, in-vehicle systems that detect and assess drowsiness and alert the driver or adjust the vehicle control if the driver is about to fall asleep are being developed. Fatigue while driving decreases attention and reduces the amount of information received by the driver. Moreover, it decreases the driver’s response and level of understanding of the situation, thus resulting in incorrect decision-making. In the worst case, the driver’s arousal level may decrease, and sleepiness might follow, which might cause loss of vehicle control. To prevent these situations, driver monitoring systems that can detect the driver’s state of drowsiness are necessary. If these systems are utilized, they can inform the driver or send a signal to the driver’s family members or friends about the drive’s low arousal level [7].

Nishiyama listed the following six conditions required for detecting drowsiness in in-vehicle systems [8]:

1. Do not interfere with the driver’s safe driving environment;
2. Can be equipped in a vehicle and withstand hard operating environments;
3. Can detect driver’s drowsiness in real-time;
4. Have a wide detection range from shallow to deep sleep;
5. Consider all drivers are detectable targets;
6. Have low cost and high scalability than other applications.

The physiological index of an in-vehicle system is determined based on whether it satisfies all the criteria. Research on the detection of drowsiness levels has spanned laboratory-level setups to in-vehicle setups; however, existing research has been able to satisfy only some of these six conditions [8]. Generally, it is difficult to detect a driver’s drowsiness because of surrounding lighting or the driver’s condition or behavioral characteristics, such as sleep apnea, talking, wearing glasses [7]. Various approaches developed to detect driver’s drowsiness have been applied to some mass-produced vehicles.

Existing approaches for detecting driver’s drowsiness, according to references [8,9], are categorized in Table 1.

In this paper, according to Table 1, the cases in which the drowsiness detection system are applied in the vehicle and the tendency of the system are discussed. In this paper, we refer to recent research trends using Google Scholar and introduce related technologies of the last couple of years. Indeed, the basic measurement methods for detecting drowsiness have not changed over the years, but in recent years, machine learning methods, especially deep learning, have been gaining attention, and more and more research is being done to apply deep learning to the detection and estimation of drowsiness. This is the reason why this review focuses on the technologies of the last few years. Since there are many cases where the technologies of companies have not been published, we introduce them based on the web survey.

**Figure 1:**
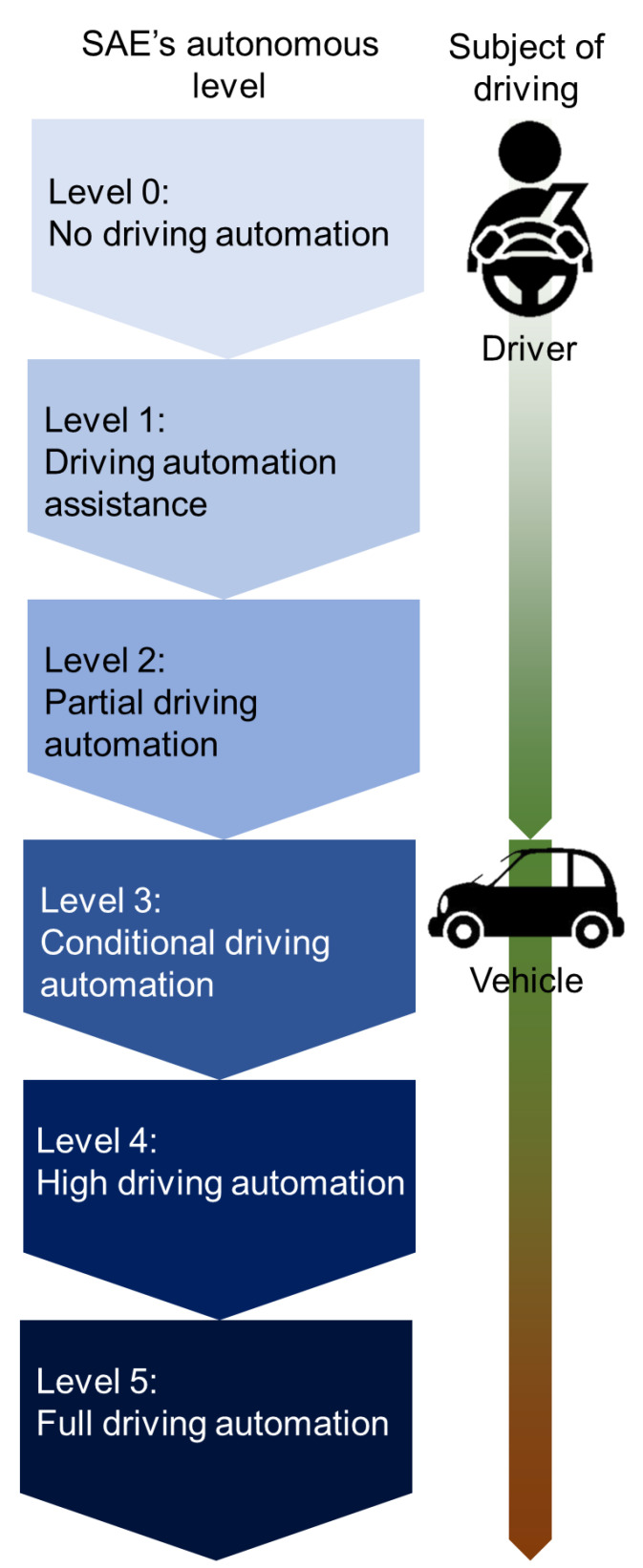
SAE’s autonomous driving level and subject of driving at each level.

The Society of Automotive Engineering defines the autonomous level [12] and the SAE’s autonomous level as the following (Figure 1) [13]:

Level 0: No driving automation—the vehicles are manually controlled;

Level 1: Driving automation assistance—the vehicles are equipped with one automated system (cruise control) for driving assistance, such as steering and acceleration;

Level 2: Partial driving automation—allow drivers to control both steering and acceleration/deceleration;

Level 3: Conditional driving automation—these vehicles have “environmental detection” features that allow them to make decisions on their own, for example, to accelerate and pass a slower vehicle, but they still need to be overridden by a human. If the system cannot perform the task, the driver must remain alert and in control at all times;

Level 4: High driving automation—in most cases, it does not require human interaction, but it can be manually overridden by a human;

Level 5: Full driving automation—it requires no human attention, not even a steering wheel or accelerator/brake pedal. It will be able to go anywhere and do anything an experienced human driver can do.

It is believed that drowsiness detection systems might no longer be applicable to vehicles driving with the Society of Automotive Engineering (SAE)’s autonomous level 4 [12]. In this paper, we discuss the development of an arousal detection system for the SAEs autonomous driving level 4 and above. The research and development of drowsiness detection systems using various methods are discussed; the applicability of such systems to autonomous vehicles is also discussed. As for the research of drowsiness detection, Rather et al. discussed fatigue and drowsiness detection techniques in driving [14]. Rather et al. categorized the characteristics of fatigue and drowsiness detection systems as “subjective reporting”, “biological characteristics”, “physical characteristics”, “vehicular characteristics”, and “hybrid characteristics” [14]. The categorization of the “biological characteristics”, “physical characteristics”, and “vehicular characteristics” is similar to that used in our study. The “subjective reporting” characteristic described by Rather et al. is only used to support the accuracy of the detection or estimation of the system, and its result is not applied to the in-vehicle system. Therefore, it is omitted from the main method. Our study differs from the study by Rather et al. because we focus on the perspective of autonomous driving. In the age of autonomous driving levels 4 and 5 proposed by SAE, the driver does not need to drive the car, so the purpose of the drowsiness detection system will change drastically, from “detecting driver drowsiness to avoid traffic accidents” to “detecting driver drowsiness to let the driver sleep peacefully and have a comfortable travel time”. Therefore, it is important and meaningful to discuss how driver drowsiness detection systems should be in the age of automated driving. This paper will contribute to this point. The classification of approaches for drowsiness detection shown in Table 1 is from 2011 and 2012; Saini and Rekha [15] and Ramzan et al. [16] present a new approach for drowsiness detection based on machine learning. These review papers are from 2014 and 2019, respectively. In the last decade, there have been significant advances in machine learning methods and in autonomous driving technology. Certainly, the review by Saini and Rekha, Ramzen et al. helps us understand the traditional and new methods of drowsiness detection. However, their review does not mention the state-of-the-art machine learning techniques, deep learning, and the emerging role of drowsiness detection systems in the age of autonomous driving. In addition, previous reviews have not considered sensor types for in-vehicle detection, but if considering in-vehicle applications, both contact and non-contact detection types should be considered, and it should be noted that non-contact drowsiness detection and estimation systems have also made remarkable progress in the last decade. In this section, we mention drowsiness detection and estimation methods using deep learning and the future potential of drowsiness detection and estimation methods in the age of autonomous driving. It also mentions the types of detection that should be considered for in-vehicle use. 

The remainder of this paper is organized as follows. Section 2 explains the purpose of the subjective evaluation of the drowsiness detection or estimation system. Section 3 explains the three evaluation methods for drowsiness detection or estimation based on biometric information, vehicle behavior, and graphic information. Section 4 discusses the prospects of in-vehicle drowsiness monitoring systems in the autonomous driving era. The conclusions are presented in Section 5.

## 2. Purpose of Subjective Evaluation for Drowsiness Detection or Estimation Systems

The validity of a drowsiness detection system cannot be detected without a standard describing how awake the driver is. Subjective evaluation is not used for such a system; however, to ensure the reliability of the system, the standard is considered a percentage of agreement between the system’s evaluation result and the driver’s level of arousal. The subjective evaluation methods described in this section were only used for the criteria to determine the reliability of the system. The Karolinska sleepiness scale (KSS) [17] and the High-frequency coupling (HFC) drowsiness scale [18] are often used as the criteria for sensory evaluation. The observer-rated sleepiness and the trained observer rating, developed by Kitajima [19,20], are similar to the HFC drowsiness scale. The trained observer rating is often used in Japan. The NEDO evaluation method [21], which is also often used in Japan, is the same as the trained observer rating. The KSS, HFC drowsiness scale, observer-rated sleepiness, and trained observer ratings have been used in many studies [22,23,24,25,26,27].

The KSS represents a subjective evaluation of sleepiness on a 9-point Likert scale. The sensory meaning of drowsiness is written on the odd-numbered rating points, and the neutral meaning is written on the even-numbered points, relative to the meaning listed on each odd-numbered rating point: 1 is “very clearly awake”, 3 is “awake”, 5 is “neither”, 7 is “sleepy”, and 9 is “very sleepy”, and, for example, 2 is between “very clearly awake” and “awake”. The reliability of the KSS for drowsy evaluation was determined in a previous study through subjective evaluation [28]; however, another study showed that the reliability of KSS is insufficient for drowsiness evaluation or estimation [25].

The HFC drowsiness scale, observer-rated sleepiness, and trained observer rating were designed to evaluate the level of arousal for which skilled evaluators were asked to apply the driver’s facial expression to the corresponding part of the scale at regular intervals using video recordings. The HFC drowsiness scale is classified into nine points, and the observer-rated sleepiness is classified into two types, D-ORS used for vehicle behavior and B-ORS used for driver behavior. Each is categorized into three scales [29], and the trained observer rating is categorized into five scales. The scale points of HFC drowsiness scale, those of D-ORS and B-ORS, and those of trained observer ratings are shown in Table 2, Table 3 and Table 4, respectively. These subjective evaluation methods are all used for drivers to evaluate their drowsiness, and the answer is used as an index to evaluate the accuracy of the system.

## 3. Drowsiness Detection and Estimation Based on Biometric Information

Drowsiness can be continuously measured using biometric information as it is an objective and direct index [6]. Electroencephalography (EEG), electrocardiography (ECG), and electrodermal activity (EDA) measurements are commonly used for evaluation [30,31]. EEG is the most used method because it is a direct neuro-activity index [32,33,34]. However, it is difficult to build an in-vehicle system that uses EEG for driver drowsiness detection and estimation because it is susceptible to noise. Moreover, electrodes are connected to the head of the person being evaluated, and the head motion is restricted; thus, EEG is used for basic consideration of drowsiness detection system, and EEG is not used for the detection methods of the system. In addition, heart rate variability (HRV) is related to the autonomic nervous system and used for drowsiness detection or estimation. The respiration and HRV were recorded and analyzed in some cases. However, it is difficult to define the direct relationship between physiological characteristics and cognitive state. These physiological characteristics do not necessarily imply only drowsiness; they also indicate other conditions, such as emotional or physical fatigue; in addition, what they reflect depends on the actual driving scenario [6]. For example, Peiris et al. showed that two professionals who analyze the EEG detect drowsiness do not necessarily make the same decision for the same participant [35]. In contrast, in some cases, the EDA is affected by stress [36] or emotion [37]. Therefore, these indicators cannot be considered solely as adequate and exclusive indicators for the detection or estimation of sleepiness or fatigue.

Satti et al. developed a system that can detect a driver’s decreasing arousal level using a steering wheel on which electrodes are attached, and electromyography was performed. They also found that as the driver’s drowsiness level increased, the muscle activity for gripping the steering decreased with time while driving. In addition, the results of the frequency domain analysis revealed that the frequency components of the steering shifted from low frequency to high frequency in an hour’s driving [38]. In one case, the driver’s states were detected by electrodes attached to the steering [39] for sensing the vitals. The study by Satte et al. is pioneering in terms of not only vital sensing but also drowsiness detection. Although no specific evaluation of drowsiness detection has been reported, Uchenna et al. performed such investigations in terms of development cases alone [40]. 

Some studies used smartwatches or smartphones for vital sensing in vehicles [41]. For example, Kindinger et al. applied machine learning only to biometric data acquired by a wearable sensor for the wrist to show the potential for driver drowsiness detection [42]. The detection results were compared with the reference data of medical ECG devices. Based on an evaluation experiment using a driving simulator with 30 participants, they found that the accuracy was comparable to that of a medical ECG and that a high accuracy (over 92%) was obtained in the “user-dependent scenario” in the driving simulator. A case study of estimating sleepiness using physiological data from a wrist-worn smart wearable device was reported [43]. In this case, a user survey (N = 30) was conducted on a test track with the SAE level 2 autonomous driving, and the heart rate data from three commercially available fitness trackers were recorded. Drowsiness was estimated using a machine learning model, and tests were conducted to classify drowsiness into binary and ternary categories. In addition, a high accuracy (over 90%) was achieved. 

## 4. Drowsiness Detection and Estimation Based on Vehicle Behavior

A typical example of mass production is a Subaru’s wobble alarm system [44]. Vehicle wobble alarms have been installed in trucks and buses, such as Hino’s Selega [45]. In recent years, Mazda has mass-produced passenger cars that make driver-attention alerts [46]. This system is activated at speeds above 65 km/h and starts learning the driver’s habits. Then, when it detects a change in the vehicle’s behavior, suggesting a loss of driver concentration, it prompts the driver to rest via information from the human–machine interface (HMI). 

Honda cars have a driver attention monitor [47]. It uses inputs from an electric power steering (EPS) to measure the frequency and degree of the driver’s steering inputs for assessing the driver’s level of awareness. The Volvo system [48] also uses lane information from cameras. Jaguar’s driver condition monitor uses the steering wheel operation status as well as brake and throttle movements [49]. There are two detection methods: one uses a steering angle sensor and the other uses a camera to detect lanes. The method based on driving behavior can estimate the driver’s level of arousal without the driver’s awareness, but it is prone to measurement errors in cases of bad weather or inadequate lane markings. In addition, it is useful only when the driver is holding the steering wheel [25].

In recent years, machine learning has been applied to detect a decrease in the arousal level. For example, Arefnezhad et al. used the steering angle as input and applied an adaptive neuro-fuzzy inference system (ANFIS) [10]. This system is useful for optimizing feature selection based on the correlation index, T-test index, mutual information index through the wrapper feature selection method. Particle swarm optimization (PSO), an evolutionary optimization method, was used to understand its parameters. An evaluation experiment was conducted on 39 bus drivers using a driving simulator. The total driving time was 53 h, and the correspondence with the sleepiness level using the KSS was evaluated. To improve the binary classification performance, a KSS of 7 was excluded. Consequently, a detection accuracy of 98.12% was achieved.

Jeon et al. proposed a new method for detecting drowsy driving using vehicle sensor data obtained from the steering wheel and pedal pressure [50]. Based on preliminary experiments, drowsy driving was classified into long-time and short-time drowsy driving types. Jeon et al. proposed an ensemble network model consisting of convolutional neural networks that could detect each type of drowsy driving. In this model, each sub-network was specialized for detecting long-time or short-time drowsy driving using the features obtained by the time series analysis. To train the proposed network efficiently, an imbalanced data handling method was adopted, which adjusted the ratio of normal and drowsy driving data in the dataset by partially deleting the normal driving data. Consequently, 198.3 h of in-vehicle sensor data were acquired through driving simulations assuming various road environments, such as urban areas and highways. Using this dataset, the performance of the proposed model was evaluated. The results showed that the proposed model was able to detect sleepiness during driving with an accuracy of up to 94.2%.

## 5. Drowsiness Detection and Estimation Based on Graphic Information of a Driver

This technique mainly uses cameras to detect the driver’s states. The Toyota Motor Corporation’s pre-crash safety system with a driver monitoring function is a relatively old example [51]. This system aims to mitigate the damage caused by a collision by issuing a warning earlier than when the driver is facing the front, specifically when it is judged that a collision is highly probable, but the driver is not facing the front. An example that is currently in operation in mass production is Toyota’s driver monitoring system (advanced drive specifications). This system uses a general-purpose processor to implement artificial intelligence (AI). It has greatly improved the detection of gaze, face direction, eyelid opening/closing. It also has a function to safely stop the car if the driver’s driving posture is distorted or the car is unresponsive to warnings [52]. Subaru’s driver monitoring system (DMS) is another example. In the case of the Forester, a camera installed on the top of the head-mounted display detects the driver’s face and warns the driver via the HMI if the eyes are closed or the driver is not focused [53]. A similar system was installed in Nissan’s skyline [54]. In commercial Hino Selega vehicles, a “driver monitor II” is installed [45]. In the past, the detection accuracy was low when glasses were worn. Recent devices have solved these problems and can detect drowsiness even with glasses worn. In recent years, external driver monitoring systems have been developed; these systems can be installed even after purchasing a car [55,56]. These systems detect the opening and closing of the eyelids or looking aside and notify the driver with a buzzer, but this does not affect the vehicle control. Babusiak et al. developed a smart steering wheel for detecting drowsiness [57]. This steering detects heart rate, heart rate variability, and blood oxygenation. In addition, an inertial unit is also integrated to record and analyze a driver’s behavior pattern.

In actual research and development, the level of arousal is estimated based on various factors related to the driver’s face by using a camera. Eyelid-related information, which can be measured using indicators such as the percentage of eyelid closure (PERCLOS) and blinks, has attracted attention because it can be measured without constraints. The NHTSA investigated the relationship between PERCLOS and other measures of arousal and reported a high correlation between them. They also investigated the relationship between PERCLOS and other indices of arousal and reported a high correlation between decreased arousal and PERCLOS [58]. For example, a previous study [59] evaluated the state as arousal when the PERCLOS value in every 3 min was 0% to 9%, suspicious when 9% to 12%, and low arousal when greater than 12%. Tanaka reported that a decrease in the arousal level increased the blink rate when the arousal level was maintained at a certain high level and that the blink rate decreased when the arousal level decreased [60]. In other words, the number of blinks increases when the subject begins to feel drowsy from a state of clear wakefulness, but it decreases when the subject becomes drowsier after the level of wakefulness decreases. However, since the PERCLOS is usually calculated from the visible images of the subject, its evaluation depends greatly on the lighting conditions, and it cannot be evaluated when the driver is wearing sunglasses [61]. Therefore, the PERCLOS was applied to visible images obtained by thermal imaging cameras and classified using machine learning [61]. In this case, the classification of “wakefulness”, “fatigue”, and “dozing” was performed, and the average classification accuracy was about 65%. There are other previous studies that use PERCLOS to detect or estimate drowsiness [62]. 

Similarly, Tashakori et al. conducted a study using temperature information [63]. They detected driver drowsiness by measuring the forehead and cheek temperatures. About 30 subjects drove on a highway in a driving simulator on two separate occasions. A thermal imaging camera was used to monitor the temperature patterns of the faces. The sleepiness level of each subject was estimated by three human observers. The support vector machine, K-nearest neighbor, and decision tree classifiers were used. From wakefulness to extreme drowsiness, the absolute values of the forehead skin temperature and cheek-forehead skin temperature gradient decreased by 0.46 °C and 0.81 °C, respectively. However, the cheek skin temperature increased by 0.35 °C over the two sessions. These results indicate that sleepiness was detected in 84% of the cases. 

Video-oculography is commonly used to study features, such as blink frequency, blink moment, and PERCLOS. It is extracted by an image-processing algorithm based on eye tracking, head, and gaze movements. Therefore, the quality of the estimation highly depends on the initial signal processing.

**Figure 2:**
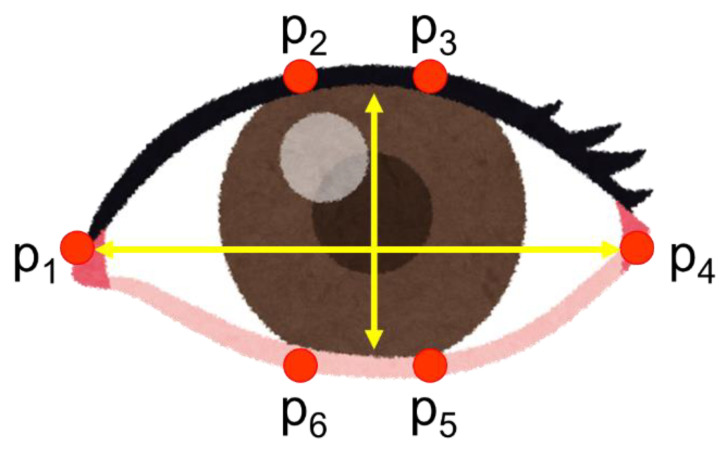
The feature points of the eye.

The eye aspect ratio is also used in some cases. When the feature points of the eye are *p*_1_–*p*_6_, the EAR is expressed by the following equation. Here, the feature points of the eye are defined in Figure 2.

$$\mathrm{EAR}=\frac{\left||p_{2}-p_{6}||+||p_{3}-p_{5}|\right|}{2||p_{1}-p_{4}||}$$

Chakkravarthy stated that in an evaluation using the EAR, the correct response rate was 75% for blinking eyes, 35% when wearing glasses, and 25% when hair was hanging over the face [64]. Suhaiman et al. implemented the system on a Raspberry Pi 3 + system such that an alarm would ring when the level of arousal decreased [65]. In this case, however, the number of subjects was small; thus, the accuracy could not be guaranteed.

Manu reported an example of drowsiness detection using facial feature detection, eye tracking, and yawn detection using a method developed by Viola and Jones [66]. When a face is detected, most of the background of the non-face image is removed based on the skin color. Then, only the skin part is segmented, and only the color component is considered to remove the effect of lighting. Eye-tracking and yawn detection are performed by template matching. The feature vectors obtained at each of the above stages are concatenated, and a binary support vector machine classifier is used to classify successive frames into fatigued and non-fatigued states. In addition, an alarm is rung when the threshold time is exceeded for the former. Experiments have shown that this method is very efficient in detecting drowsiness and alerting drivers [67].

Selvakumar et al. proposed an eye state classification method based on partial least squares (PLS) analysis and its real-time implementation on a resource-constrained digital video processor platform to monitor the eye state while driving [68]. Drowsiness was detected using PERCLOS. In this method, a cascade classifier based on Haar features was used to detect faces in an infrared image, and the eyes were detected among the faces. To classify the eyes in a binary manner, PLS analysis was applied to obtain a low-dimensional discriminative subspace, in which a simple PLS regression score-based classifier was used to classify the test vectors into open and closed eyes. In in-vehicle testing, they claimed that the proposed system achieved a significant improvement in the classification accuracy at approximately three frames per second.

Nowadays, the application of deep learning for detecting and estimating drowsiness in vehicle seems to be remarkable, and we can say that the use of deep learning is important and essential to develop drowsiness detection and estimation technology because deep learning can handle tremendous data and features. In a case study of estimating arousal from mouth movements, Li et al. introduced an improved YOLOv3-tiny convolutional neural network to capture facial regions under complex driving conditions, thereby eliminating the inaccuracies and effects caused by artificial feature extraction. The eye feature vector (EFV) and mouth feature vector (MFV), which are the evaluation parameters of the driver’s eye and mouth states, were introduced based on the Dlib toolkit. Then, through offline learning, a driver ID information library, including a classifier library for the driver’s eye state, a classifier library for the driver’s mouth state, and a biometric library for the driver, was constructed. Finally, driver identification and fatigue evaluation models were constructed via online evaluation. The driver’s eye closure time, the number of blinks, and the number of yawns were calculated to evaluate the fatigue state. As a result, the fatigue state could be detected with 95.10% accuracy [69]. Képešiová et al. obtained grayscale facial image data of 20 collaborators and trained them using the convolutional neural network (CNN). Celecia et al.’s case study combined measures of sleepiness based on information about the eyes (PERCLOS, eye closure time) and mouth free time via a fuzzy inference system on a Raspberry Pi system to enable real-time responses [70]. The system detected three drowsiness levels (low/normal, medium/drowsy, and high/severe drowsiness), which were evaluated in terms of their computational performance and efficiency, resulting in a significant accuracy of 95.5% in state recognition [62]. Dua et al. used four deep learning models, AlexNet, VGG-FaceNet, FlowImageNet, and ResNet, to detect sleepiness by considering four different types of features: hand gestures, facial expressions, behavioral features, and head movements. These models were classified into four classes of output: “not sleepy”, “sleepy blinking eyes”, “yawning”, and “nodding”. The accuracy obtained using this system was 85% [71].

In another study, although a camera was not used, nodding was detected using a long short-term memory (LSTM) autoencoder for data from a radio frequency identification (RFID) tag attached to the back of a hat, and fatigue and decreased alertness were estimated [72].

## 6. Combining Multiple Types of Data

In many of the cases mentioned so far, deep learning has been applied to a single type of data. It is particularly used in cases where image processing is involved. However, with the rapid emergence of deep learning, it has become possible to handle various types of data simultaneously, and accuracy can be guaranteed. For this reason, in recent years, there have been few cases of estimating wakefulness using only vehicle and biometric information but several cases of estimating wakefulness using deep learning methods in combination with this information and image data. In particular, there might be many combinations, such as “vehicle information + biometric information”, “vehicle information + image information”, and “vehicle information + biometric information + image information”.

First, an example of using an artificial neural network to detect sleepiness [6] is introduced here. In this case, multiple indicators (driver physiology, psychology, behavior, and vehicle behavior) were used. Different datasets with different sources of information were tested to determine the indicators that produced the most powerful models. In this example, two hypotheses were formulated. The first hypothesis is that the sensory-motor, physiological, and performance measures used to detect sleepiness can be used to predict when a drop in sleepiness occurs, and the second hypothesis is that adding information, such as the driving time and participant information, improves the accuracy of the model.

Twenty-one participants (11 male and 10 female) participated in the experiment. They drove for 90 min on the expressway, 5 min on the route to the city, and 5 min in the city. Twenty-two cars appeared from the right side of the expressway and disappeared after a few kilometers to change the driver’s sleepiness level after 2/3rd of the route. 

The model with the highest detection and prediction accuracy was the one that used the information of eyelid closure, eye and head movements, and driving time. This model was able to predict when the driver’s state would become impaired within 5 min, and the prediction performance was promising. Furthermore, by modeling drowsiness as a continuous value, it was possible not only to detect whether a driver is drowsy but also to develop a more precise detection system.

In a previous study [23], 71 subjects (48 male, 23 female) were included. In another previous study [23], 71 subjects (48 male and 23 female; age range 22–60 years; mean age 38 years; standard deviation 10 years) were tested using a driving simulator, and the results showed a correct response rate of 72.7% in three classifications (awake, suspicious drowsiness, and sleepy). In this study, the eye closure index was used. This index is expressed as follows.

$$EyeClosure=max\left(1-\frac{d}{d_{I}},\;0\right)$$

**Figure 3:**
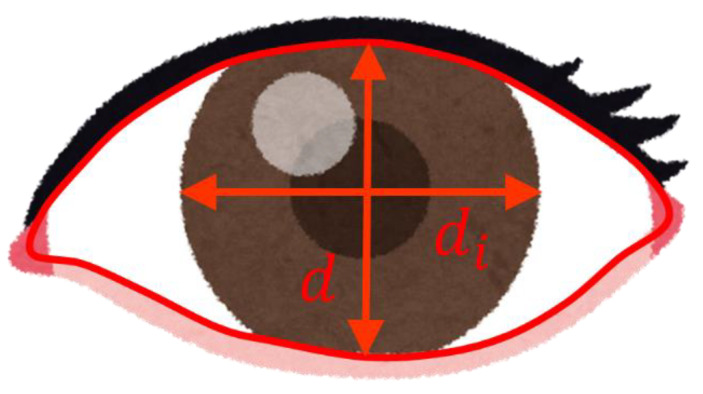
Definition of d and $d_{i}$.

Here, d and $d_{i}$ is defined in Figure 3.

Logistic regression was used with eye closure and head movement, KSS, the incidence of the HFC and lane departure, and lateral standard deviation as explanatory variables. Fatima et al. used TensorFlow, Python3, and OpenCV to detect sleepiness in real-time [73]. They used yawning, prolonged eye closure, reduced driver movement, lane change, and oversteering for training. If these data indicate that the driver is not paying attention, the alarm sounds in the car, and the driver’s seat begins to vibrate. If the driver does not respond, the system takes measures such as gradually slowing down the car. Jabbar et al. [74] developed a drowsiness detection system based on a multi-layered perceptual classifier, intended to be embedded in Android mobiles and other devices, which can detect facial landmarks to identify the driver’s state. The accuracy of this system is very good. The accuracy of this system is said to be 81%, including with or without glasses, and the state of night or not. Ma et al. [75] introduced a modified hierarchical extreme learning machine algorithm with particle swarm optimization (PSO-H-ELM) and classified the driver’s drowsiness by the power spectrum density based on EEG data, and they showed the accuracy is 83.12%. Ariizumi et al. developed the estimation algorithm by echo state network (ESN) [76]. ESN is a kind of Recurrent Neural Network (RNN), which is a neural network adapted for processing time-series data and a kind of deep learning. ESNs are characterized by their low computational cost, although the impact of computational cost must be considered when implementing deep learning. In this case study, 40 male and female collaborators in their 20s to 60s drove on a highway-like environment for about one hour using a driving simulator. Pulse, respiration, and center of gravity information were obtained as physiological indicators of the drivers. In addition, drivers were asked to self-report their sleepiness every two minutes on a five-point scale. In addition, the driver’s face image was recorded as a movie, and a third party judged the driver’s sleepiness based on the NEDO evaluation method. The actual level of drowsiness was determined by the greater of the self-reported result and the result of the NEDO evaluation method. Therefore, in this case, the physiological indices of the driver were used as explanatory variables, and the ESN was used to estimate the actual sleepiness level of the driver.

The actual drowsiness level was estimated by ESN after converting the level 2 or lower into a binary value of “not sleepy” and the level 3 or higher into “sleepy”. The correct response rate was 83.3%, the true positive rate (when the ESN estimated that the driver was also sleepy when the driver was actually sleepy) was 88.7%, and the true negative rate (when the ESN estimated that the driver was also not sleepy when the driver was actually not sleepy) was 63.2%. The performance was relatively high when converting to binary values and estimating “drowsy” and “not drowsy” in this way. On the other hand, when the original 5-step evaluation was utilized as is and 5-step estimation was performed, the existence of time periods in which the estimation of sleepiness changed frequently and a discrepancy between the actual and estimated values were observed, suggesting that improvements are needed. However, considering the fact that there are large individual differences in the way sleepiness appears in biological signals, we believe that the estimation accuracy is quite high for a judgment without tuning to suit each individual.

## 7. Summary of Current Technology Trends

Table 5 summarizes the technologies reviewed in this paper. To the best of the author’s knowledge, the specific detection accuracy of the systems used by manufacturers has not been disclosed, probably due to patent and confidentiality restrictions. With regard to the detection and estimation methods discussed in this study, it is considered that the agreement rate with the level of arousal based on subjective evaluation is approximately 70% to 90% while being dependent on the number of collaborators and the environment. If a specific method is established, it is likely to be used for mass production. However, no particular method is currently being used in wakefulness detection and estimation technology. However, with the recent use of deep learning, if in-vehicle computers and GPUs can be installed and data from a large number of drivers can be obtained, considerable accuracy can be guaranteed regardless of the method used. Therefore, research advancements achieved using deep learning will help determine the technology for detecting and estimating wakefulness in the future. However, since there have been very few examples of evaluations using actual vehicles, it is believed that the effects of the environment, such as ambient light, road surface vibrations, and individual differences among drivers, have not been taken into account. 

## 8. Arousal Level Detection and Estimation Technology for Autonomous Driving

The technologies for detecting and estimating the level of alertness described so far are useful for SAE autonomous driving level 3 or lower, where the authority of the driver is guaranteed. However, in the SAE autonomous driving levels 4 and 5, the driver is not involved in driving the vehicle. However, it may be possible to use arousal detection and estimation technology to ensure that the driver sleeps comfortably until the destination. According to a questionnaire, about 34% of the respondents answered that it is acceptable to sleep in an autonomous vehicle, and 12.7% answered that they were okay with sleeping, and it seems that almost 50% of the respondents were okay with sleeping in an autonomous vehicle [77]. Therefore, for example, by using arousal detection and estimation technology, when the driver feels drowsy, the seat can be folded down, and the driver can be induced to sleep. For example, technology to eliminate drowsiness is being developed by having the driver sleep comfortably for a short period while driving automatically [78]. In addition, the use of technologies that realize a pleasant awakening by using sound, light, and scent when the driver is awake [79,80,81] could allow the driver to sleep comfortably when sleepy and to wake up pleasantly; this can enable the driver to reach the destination without feeling any physical or mental burden. In this case, however, because the driver does not need to drive, vehicle information is not used as an indicator of decreased wakefulness, and the system is mainly based on the biometric information or driver behavior. In addition, even if the accuracy of the arousal detection is poor, it can be used to ensure driving safety. In the current SAE autonomous driving level 0 to 3, if accuracy is not ensured, it does not lead to accident prevention; this could be dangerous in terms of the use of the system and the transfer of authority between the driver and the car in autonomous driving level 3. However, if automatic driving levels 4 and 5 are realized, even if the detection is delayed, it does not affect the driving itself but could only make a difference in the driver’s comfort; therefore, it can be applied even if the accuracy is not guaranteed. Therefore, while improving the accuracy of the current arousal detection and estimation system is a priority, a system can be developed to provide comfort in a situation of autonomous driving (SAE levels 4 and 5). 

Table 6 summarizes the prospects of arousal level detection and estimation technology for autonomous driving, as shown above.

## 9. Summary

In this study, the current mass-produced technologies and main detection and estimation methods used for arousal detection and estimation were summarized, and the prospects for arousal detection and estimation technologies in levels 4 and 5 autonomous driving were discussed. Regarding future technologies for detecting and estimating wakefulness, the cost of the system in which they are installed will depend on the cost of the component sensors; however, if the use of machine learning (deep learning) is promoted by the installation of computers and GPUs, the accuracy of the system can be improved, and the installation of such systems in vehicles is expected to increase. However, an evaluation in an actual vehicle environment is necessary. Moreover, in the case of autonomous driving levels 4 and 5, where the human driver is not the primary driver, the technology need not be used to detect and estimate drowsiness in the sense of accident prevention. Therefore, the drowsiness detection and estimation systems in the era of autonomous driving levels 4 and 5 may lead to drivers feeling more comfortable in their cars. In such a case, the drowsiness detection and estimation system become an application to detect the driver’s drowsiness and put the driver to sleep. The system will then help the driver sleep comfortably and arrive at their destination comfortably. In other words, in the future, the drowsiness detection and estimation system will function as a system that brings comfort when traveling so that the driver can reduce the fatigue associated with traveling when using an automobile and can act without feeling tired after arriving at the destination. Drowsiness detection and estimation system for autonomous driving levels 4 and 5 will be applied not only to automobiles but also to other fields and industries related to sleep in the future, in terms of detecting drowsiness and making people feel comfortable. 

## Figures and Tables

**Table 1:** Approaches for detecting driver’s drowsiness.

Methods	Measurement Information	Measurement Target	Measurement Method	Measurement Index	Advantages	Disadvantages
Contact method	Biometric information	Heartbeat, pulse wave, aspiration, brain wave, myoelectric, eye movement, etc.	Heart rate monitor, pulse wavemeter, electroencephalograph, electromyograph, nystagmus, etc.	Heart rate, chaos analysis, alpha wave, theta wave, muscle action potential, vestibular oculomotor reflex, etc.	High drowsiness detection performance can be obtained [10].	Driver behavior adversely affects the reliability of the designed system [10].
Non-contact method	Vehicle behavior	Steering pattern, distance between lane and vehicle, speed, distance between vehicles, etc.	Steering angle sensor, white line recognition camera, laser radar, etc.	Steering frequency, meandering rate, steering volume, monotonous steering	Estimates can be obtained in a way that is unobtrusive to the driver [11].	The accuracy of detection and estimation depends on road conditions and the environment. It is useful only when the driver is holding the steering wheel.
	Driver’s graphic information	Open rate of eyes, blink, pupil, voice, and expression	Camera, microphone	Opening and closing rates of eyes, number of blinks, time of closing eyes, pupil fluctuation, chaos analysis of speech sound, drowsy categorization by expression, etc.	Intuitional index and easy-to-understand, high accuracy.	Different lighting conditions may disrupt the detection performance [10].

**Table 2:** HFC drowsiness scale.

HFC	Description
1	Wide awake, vivid attention
2	Highly concentrated, focused attention
3	Attentive but calm
4	No activation, no drowsiness, no pronounced tendency for reactive behavior
5	Slightly dozing, ready to respond
6	Signs of drowsiness but effortlessly awake
7	Obvious drowsiness, but mainly focused on driving tasks
8	Battling with drowsiness. Difficulty with driving tasks, but mainly perceptual
9	Feeling foggy, listless, inactive for long periods of time, microsleep is occurring or may be occurring

**Table 3:** D-ORS and B-ORS.

D-ORS0 (Alert)	B-ORS0 (Alert)
Awareness: driver’s reactions are high and fast Driving: normal	Blink: normal Yawning: no Body position: sitting still Body movements: hardly
D-ORS1 (First signs of sleepiness)	B-ORS1 (First signs of sleepiness)
Awareness: driver’s reactions are relatively normal and fast Driving: light steering wheel operation	Blink: sporadic prolonged closure of the eyelids, followed by increased blinking frequency Yawning: occasionally Body position: sometimes change position Body movements: sometimes
D-ORS2 (Severe sleepiness)	B-ORS2 (Severe sleepiness (microsleep))
Awareness: driver reacts slowly Driving: cannot drive steadily and turns the steering wheel too far	Blink: driver’s eyes are half-closed, and his/her gaze vacant Yawning: frequently Body position: frequently change Body movement: frequently

**Table 4:** Trained observer rating.

Level	Phenomenon
1	Do not look sleepy at all; gaze moves quickly and frequently, blink at a constant rate of about 2 times every 2 s, and body movements are active.
2	Slightly sleepy, open lips, slow eye movement.
3	Looks somewhat sleepy, blinks slowly and frequently, mouth moves, sits up straight, and puts hands on face.
4	Looks quite sleepy and blinks as if conscious. Unnecessary movements of the entire body, such as shaking the head or moving the shoulders up and down. Frequent yawning and deep breathing. Slow blinking or eye movements.
5	Looks very sleepy, eyelids closed, head tilted forward or back.

**Table 5:** Summary of the technologies discussed in this paper. NA means that the accuracy could not be ascertained within the reference.

Methods	Measurement Information	Previous Studies	Method	Accuracy
Contact method	Biometric information	Satti et al. [38]	Electromyogram measurement from electrodes attached to the steering wheel Electrocardiographic measurements from a wearable sensor on the wrist.	NA
		Kundinger et al. [42]		≧92%
		Kundinger et al. [43]		≧90%
Non-contact method	Vehicle behavior	Subaru [44] Hino [45] Mazda [46] Honda [47] Volvo [48] Jaguar [49]	Detects changes in vehicle behavior and warns from HMI.	NA
		Arefnezhad et al. [10]	Apply ANFIS with steering angle as input.	98.12%
		Jeon et al. [50]	Estimation by ensemble network model using steering and pedal pressure as input.	94.2%
	Graphic information (of driver)	Toyota [51] Subaru [53] Nissan [54] Hino [45] Thanko [55] Yupiteru [56]	Warnings for closed eyes and side glances.	NA
		Toyota [52]	Stops the car when the driver is not in a good posture or does not respond to warnings.	
		Cardone et al. [61]	Applied PERCLOS to visible images obtained by a thermal imaging camera and classified “wakefulness”, “fatigue”, and “dozing” by deep learning. Support vector machine, K-nearest neighbor method, and decision tree were used to classify sleepiness based on the temperature patterns of the forehead and cheeks.	Approximately 65%
		Tashakori et al. [63]		84%
Non-contact method	Graphic information (of driver)	Celecia et al. [62]	Fuzzy inference system to estimate sleepiness from eye and mouth information.	95.5%
		Chakkravarthy [64]	EAR	75% when blinking, 35% when wearing glasses, and 25% when hair is hanging over the face
		Manu [67]	Correlation coefficient template matching.	94.58%
		Li et al. [69]	Detecting fatigue from driver’s eye closure time, few blinks, and few yawns.	95.10%
		Képešiová et al. [70]	Learning grayscale face images with CNN.	98.02%
		Dua et al. [71]	Detects drowsiness by considering four different types of features (hand gestures, facial expressions, behavioral features, and head movements) using four deep learning models: AlexNet, VGG-FaceNet, FlowImageNet, and ResNet.	85%
		Yang et al. [72]	Nodding detection using LSTM autoencoder on RFID tag data.	≧90%
		Jabber et al. [74]	Facial landmarks from images were detected and estimated by a system based on multilayers perception classifiers.	81%
		Ma et al. [75]	Classified the driver’s drowsiness by PSO-H-ELM based on the power spectrum density of EEG data.	83.12%
Multiple methods		de Naurois et al. [6]	Modeled using the information on eyelid closure, eye and head movements, and driving time. Logistic regression with Eye Closure, head movement, KSS, HFC, etc., as explanatory variables.	MSE of drowsiness level: 0.22
		Baccour et al. [27]	Pulse, respiration, and center of gravity information were obtained, and ESN was used for estimation.	72.7%
		Ariizumi et al. [76]		83.3%

**Table 6:** Summary of the prospects of arousal level detection and estimation technology for autonomous driving.

SAE’s Autonomous Driving Level	Purpose of the Technology
0, 1, 2 and 3	Detect and estimate the driver’s drowsiness and notify the driver of the result to prevent human error caused by drowsiness.
4 and 5	Detect and estimate the driver’s drowsiness and makes the driver sleep so that they can comfortably reach the destination.
